# Motivational Non-directive Resonance Breathing as a Treatment for Chronic Widespread Pain

**DOI:** 10.3389/fpsyg.2019.01207

**Published:** 2019-06-11

**Authors:** Charles Ethan Paccione, Henrik Børsting Jacobsen

**Affiliations:** ^1^Department of Pain Management and Research, Oslo University Hospital, University of Oslo, Oslo, Norway; ^2^Department of Pain Management and Research, Oslo University Hospital, Oslo, Norway

**Keywords:** chronic widespread pain, non-directive meditation, diaphragmatic breathing, transcutaneous vagal nerve stimulation, baroreceptor sensitivity, heart rate variability, resonance frequency breathing, motivation

## Abstract

Chronic widespread pain (CWP) is one of the most difficult pain conditions to treat due to an unknown etiology and a lack of innovative treatment design and effectiveness. Based upon preliminary findings within the fields of motivational psychology, integrative neuroscience, diaphragmatic breathing, and vagal nerve stimulation, we propose a new treatment intervention, motivational non-directive (ND) resonance breathing, as a means of reducing pain and suffering in patients with CWP. Motivational ND resonance breathing provides patients with a noninvasive means of potentially modulating five psychophysiological mechanisms imperative for endogenously treating pain and increasing overall quality of life.

## Introduction and Background

Chronic widespread pain (CWP), including fibromyalgia syndrome (FMS), is a particular type of chronic primary pain where biological causalities may or may not be present and can not be identified as either musculoskeletal or neuropathic pain (Treede et al., 2015). CWP is a multifactorial pain condition characterized by prolonged pain that lasts for 3 months or more in multiple regions of the body. It is often associated with significant psychophysiological distress in the form of anxiety, anger, frustration, depression, insomnia, and social isolation (Maletic and Raison, 2009; Mansfield et al., 2017; World Health Organization, 2018). CWP is estimated to have a global prevalence of about one in every ten adults (Mansfield et al., 2016; Andrews et al., 2017) with a societal cost more than that of cancer and diabetes combined (Vos et al., 2013). Among all of the chronic pain conditions today, CWP is one of the most difficult to treat and manage (Lee et al., 2014).

Many of the symptoms associated with CWP overlap with those of functional somatic syndromes (FSS) (Henningsen et al., 2007) and medically unexplained symptoms (MUS) (Konnopka et al., 2012) where pathologically unexplainable patterns of persistent bodily complaints are present, some of which include pain in various locations, functional disturbances in different organ systems, and complaints centering around physical exhaustion and mental fatigue (Loeser and Melzack, 1999). Currently available treatments for FSS or MUS conditions provide modest improvements in pain and minimal improvements in both physical and emotional functioning (Turk et al., 2011). The difficulty to manage CWP in particular may be due to an unknown etiology (Sommer et al., 2008), an unstandardized definition (Butler et al., 2016), and a lack of both mainstream and alternative treatment modalities that are specifically designed to meet the psychophysiological profile of those suffering from it (Bee et al., 2016). General practitioners still have difficulty recognizing CWP and FMS as a valid diagnosis and often have limited awareness of diagnostic criteria and clinical models that may describe its psychosomatic interface (Mansfield et al., 2017).

Interdisciplinary theories which include the neurovisceral integration model (Thayer, 2007, 2009), the polyvagal theory (Porges, 2009; Kolacz and Porges, 2018), the biological behavioral model (Grossman and Taylor, 2007), the resonance frequency (RF) model (Lehrer, 2013), and the psychophysiological coherence model (McCraty and Childre, 2010) have all proposed that vagal tone (i.e., parasympathetic activity) is an arbiter for nurturing both psychological and physiological wellbeing. The neurovisceral integration model states that high vagal tone leads to better cognitive and emotional functioning as well as health regulation (Thayer, 2009). The polyvagal theory states that high vagal tone also leads to better social functioning (Kolacz and Porges, 2018). The biological behavioral model (Grossman and Taylor, 2007) understands vagal tone as a mediator and regulator of energy exchange through the synchronization of respiratory and cardiovascular processes during metabolic and behavioral changes. This model understands high vagal tone as a means of adaptation. In order to increase vagal tone, the RF breathing model was put forth which states that slow paced breathing at RF can increase vagal tone. Lastly, the psychophysiological coherence model (McCraty and Childre, 2010) takes it one step further by postulating that slow-paced breathing practiced with positive emotions can increase personal, social, and global health. Even though these theories share a common notion that vagal tone may be an important factor to consider when optimizing psychophysiological health, they lack a formalized means of methodologically applying the theory to practice within the field of CWP research and management.

Our unified theoretical approach herein referred to as the *motivational nondirective resonance model* attempts to bridge the aforementioned theories and describe a mechanism-based interventional approach, motivational non-directive (ND) resonance breathing, for treating CWP. To our knowledge, we are the first to propose such a unified theory and describe how it can be applied and tested as an innovative treatment for CWP. Interventions which have been previously developed in order to apply these theoretical findings to the development and delivery of new pain treatments have failed to sufficiently treat CWP.

None of the most commonly used pharmacological, psychological, medical, or surgical treatments are, by themselves, sufficiently able to remove pain or to significantly enhance physical and emotional functioning for patients suffering from CWP (Turk et al., 2011). However, primary care physicians continue to treat persistent pain conditions with chronic opioid therapy (Turner et al., 2016) despite the fact that opioids generally fail to alleviate pain intensity and function (Sullivan et al., 2008; Boudreau et al., 2009; Chou et al., 2015) and cause a myriad of adverse side effects (Ivanova et al., 2013; National Academies of Sciences Engineering and Medicine, 2017). This may be a reason as to why nearly half of those diagnosed with CWP/FMS still receive inadequate pain relief and often report partial or whole work incapacity (Breivik et al., 2006), increased sick-leave, poor quality of life and health (Gerdle et al., 2008; Mayer T.G. et al., 2008), and continue to suffer from a wide variety of psycho-social issues (Järemo et al., 2017).

Non-pharmacological treatments for FSS and MUS that involve the active participation of patients, such as exercise and psychotherapy, have been shown to be more effective than treatments which only involve passive physical measures (i.e., injections and operations) (Henningsen et al., 2007). Therefore, many patients decide to seek psycho- behavioral treatment plans that include adjunctive therapy or alternatives to medication (Chiesa and Serretti, 2011). A complementary and alternative medicine (CAM) survey report (Barnes et al., 2008) found that 4 out of 10 United States adults had used CAM therapy in the past 12 months. One of the most commonly used CAM therapies is deep breathing exercises for treating back pain, neck pain, and joint pain — all common symptoms inherent in those diagnosed with CWP (Barnes et al., 2008; Häuser et al., 2017). However, treatment modalities such as cognitive behavioral therapy, acceptance and commitment therapy, and mindfulness-based therapies — all of which commonly employ training modalities in intra and interpersonal psychology, deep breathing, positive affect, and executive control — have overall weak to moderate effect sizes for treating CWP when compared to treatment as usual, passive controls, and/or educational support groups (Morley et al., 1999; Hofmann and Asmundson, 2008).

In particular, research on mindfulness-based meditation interventions show contradictory findings (Farias et al., 2016), differences in conceptualization and practice (Chiesa and Malinowski, 2011), positive report biases (Coronado-Montoya et al., 2016), and only small to moderate effect sizes for treating pain in clinical populations (Veehof et al., 2011; Williams et al., 2012). Due to these findings, CWP continues to pose a tremendous burden on society and individuals (Hilton et al., 2016) who are searching for effective and integrative means of treatment. The overall inadequacy of mainstream and alternative treatments illustrates the necessity to develop innovative approaches for safely and effectively treating the specific psychophysiological framework of CWP. One promising avenue for treating CWP may be through the manipulation of respiratory mechanics.

Subsequent data shows a strong bidirectional relationship between pain and respiration (Perri and Halford, 2004; Smith et al., 2006; Jafari et al., 2017). Pain can cause faulty respiration (such as hyperventilation and breath-holding) which has a stronger association with chronic low back pain than obesity and physical activity (Borgbjerg et al., 1996; Nishino et al., 1999; Kato et al., 2001; Smith et al., 2006). Clinical studies demonstrate that deep breathing techniques may have positive effects for treating acute pain conditions (Jafari et al., 2017). However, the positive analgesic effects deep breathing may have on some acute pain conditions has failed to be established for CP conditions such as CWP. Experimental evidence elucidating the underlying psychophysiological mechanisms of how deep breathing may be used to treat CWP is lacking and often inconsistent (Jafari et al., 2017).

Due to the strong bidirectional relationship between pain and respiration, a recent systematic review (Jafari et al., 2017) called for future research to identify the autonomic and cardiovascular mediators that link respiration and pain; identify the physiological (i.e., respiratory) mechanisms needed to reduce pain; identify the central mechanisms responsible for producing respiratory hypoalgesia; and identify the psychological (i.e., behavioral) mechanisms needed to reduce pain.

In this paper, we propose that the autonomic and cardiovascular mediators that link respiration and CWP are baroreceptor sensitivity (BRS) and heart rate variability (HRV); the physiological mechanism needed to reduce pain in those with CWP is diaphragmatic breathing (DB) (i.e., RF breathing); the central mechanism responsible for producing respiratory hypoalgesia is vagus nerve stimulation; and the cognitive and affective psychological mechanisms needed to reduce pain in those with CWP is ND attention and motivation.

We believe that DB practiced at one’s RF has the potential to increase HRV and BRS by stimulating the vagal nerve in those suffering from CWP. Indeed, stimulation of the vagus nerve targets several pathophysiological factors associated with CWP. If diaphragmatic RF breathing is practiced with a ND quality of attention, we believe that this may be the ideal means of modulating specific brain activity (i.e., the default mode network) and thus remodel the relationship between CWP and emotion. Furthermore, motivating CWP patients to practice ND diaphragmatic RF breathing everyday while increasing their positive treatment expectations may aid in targeting the immune-mediated parameter of CWP. We hypothesize that motivational ND resonance breathing (MNRB) is a potential psychophysiological intervention for endogenously treating pain intensity and disability in those who suffer from CWP.

## Cardiovascular and Autonomic Mediators: Baroreceptor Sensitivity and Heart Rate Variability

Baroreceptor sensitivity and heart rate variability are among the most important factors for evaluating the health and functionality of the cardiovascular and autonomic systems in those suffering from CP (Bruehl et al., 2018). BRS is a measure of the baroreflex, a homeostatic negative feedback loop important for maintaining healthy constant blood pressure levels in accordance with the requirements of a given situation (Mason et al., 2013). Changes in BRS may be involved in modulating the activity of endogenous pain modulatory systems (Kamibayashi and Maze, 2000). Recent research has also suggested that BRS may play a key role in the relationship between cardiovascular, respiratory activity, and pain dampening through the cardiovascular or central branches of the baroreceptor system (Jafari et al., 2017). HRV represents the change in the time interval between successive heartbeats and is used as an index of cardiac vagal tone (also known as cardiac vagal control), which is the contribution of the parasympathetic nervous system (i.e., vagal tone) to cardiac regulation (Brodal, 2004; Nahman-Averbuch et al., 2016; Laborde et al., 2017).

Among all of the time- and frequency-domain HRV parameters, the standard deviation of NN intervals (SDNN), the percentage of successive RR intervals that differ by more than 50 ms (pNN50), the high- frequency power (hf), and the root mean square of successive RR interval differences (RMSSD) are considered to reflect cardiac vagal tone (Telles et al., 2016; Laborde et al., 2017). However, due to their strong correlation (Kleiger et al., 2005) and ability to index self-regulation at the cognitive, emotional, social, and health levels (Thayer et al., 2009; McCraty and Shaffer, 2015), both RMSSD and hfHRV (specifically between 0.15 and 0.40 Hz) (Laborde et al., 2017) are considered the most optimal parameters for measuring cardiac vagal tone (Thayer and Lane, 2000).

The largest and broadest population study to date (Bruehl et al., 2018) has shown that beyond the effects of age, sex, and body mass index, those with CP have overall lower BRS and lower HRV in both the time domain (SDNN and rMSSD) and in the frequency domain (hfHRV) when compared to pain-free controls. In particular, those with CWP have a significantly lower BRS when compared to healthy subjects without CWP; persistent stress, pain behavior, and classical and operant conditioning mechanisms can all contribute in reducing BRS in those with CWP (Chung et al., 2008). Moreover, the inverse relationship between resting BP and pain sensitivity in healthy subjects becomes impaired in those with CWP (Meller et al., 2016). Instead of diminishing central sensitization and enhancing descending pain inhibition, elevated resting BP in those with CWP increases central sensitization and weakens descending pain inhibition. In turn, this can increase pain intensity (Coderre and Melzack, 1987; Chung and Bruehl, 2008).

A large study (Barakat et al., 2012) has also shown that there is a strong association between high pain intensity and low parasympathetic tone (as indicated by lower SDNN and lower RSA) for those with CWP when compared to healthy controls without CWP. The fact that low parasympathetic tone is not associated with the presence of CWP, but instead associated with high pain intensity, suggests that the experience of intense pain is a chronic stressor interfering with parasympathetic activity (Geenen and Bijlsma, 2010; Barakat et al., 2012; Evans et al., 2013; Pittig et al., 2013).

Pioneering research within the field of respiratory hypoalgesia has shown that DB performed with a high respiratory volume and low frequency could activate the anti-nociceptive effects of BRS (Dworkin et al., 1979; Dworkin et al., 1994) and concomitant increases in hfHRV (Triedman and Saul, 1994; Bruehl and Chung, 2004). This is in line with current evidence which does not support a direct causal association between DB and pain reduction, but instead, strongly suggests that a more indirect mediation through autonomic and cardiovascular changes is plausible (Jafari et al., 2017). We believe that an indirect mediation of pain through changes in HRV (autonomic) and BRS (cardiovascular) may be the most effective and plausible way DB can lower pain intensity and disability for those with CWP.

## Physiological Mechanism: Diaphragmatic Resonance Frequency Breathing

Diaphragmatic Breathing (DB) is a pattern of expiration and inspiration in which most of the ventilatory work is executed by the diaphragm (Cahalin et al., 2002; Mosby, 2009). The diaphragm is a large, dome-shaped muscle located at the base of the lungs and is considered the most efficient muscle of breathing (Maitre et al., 1995; Cleveland Clinic, 2018a). DB is typically practiced by either laying in the supine position or sitting comfortably in a chair; the practitioner is instructed to emphasize a slow deep outward abdominal movement during inspiration and a slow deep inward abdominal movement during expiration (Cancelliero-Gaiad et al., 2014; Cleveland Clinic, 2018a). Factors which determine the physiological response of DB on pain are typically breadth frequency (breadths per minute) (Raghuraj et al., 1998; Park et al., 2013) and breadth volume (as indicated by respiratory depth) (Jafari et al., 2017).

The normal respiratory frequency for an adult at rest is around 12 to 20 breaths per minute whereas the respiratory frequency for performing DB can range from 5 to 8 breaths per minute (Shannahoff-Khalsa and Kennedy, 1993; Cleveland Clinic, 2018b). Many studies which have investigated DB for the treatment of CWP have been unclear in regard to what respiratory frequency patients should perform (Lehrer et al., 2000; Mohammed and Mohammed, 2014; Celhay et al., 2015; Jafari et al., 2017). Due to the significant relationship between low HRV and high pain intensity in CWP (Barakat et al., 2012), a DB frequency that can yield the greatest increase in hfHRV would be ideal. Experimental studies with healthy subjects (Pal and Velkumary, 2004; Jafari et al., 2017; Steffen et al., 2017) suggests that performing DB at a rate of around 6 breaths per minute (i.e., 0.10 Hz) may yield significant analgesic effects.

Breathing at a rate of 6 breaths per minute causes spontaneous oscillations in blood pressure (BP) to synchronize with BP oscillations caused by DB (Jafari et al., 2017). In turn, this can cause heart rate (HR) and breathing to synchronize, also known as RF breathing. The most common RF breathing rate is 5.5 or 6 breaths/min (Vaschillo et al., 2002) however, each person may have a unique RF breathing rate that typically ranges between 4.5 and 7.0 breaths/min. As people slow their breathing down and approach RF, the highest levels of HRV are typically obtained (Courtney et al., 2011; Steffen et al., 2017). Maximal fluctuations in HR (hfHRV) causes an increase in BP and BRS (Lehrer et al., 2003; Lagos et al., 2008).

Harmonic coupling between HRV, respiration, peripheral BP, and skin blood flow in a 0.15 Hz rhythm band (range: 0.12–0.18 Hz) has been demonstrated in healthy long- term practitioners of autogenic training (AT), a practice which uses visual imagery, body awareness, and DB exercises to promote a state of deep relaxation (Perlitz et al., 2004). In regard to pain treatment, medium-range positive effect sizes of AT and of AT versus control in a meta-analysis were found for several common symptoms and conditions inherent in those with CWP, some of which include somatoform pain disorders (unspecified type), anxiety disorders, and functional sleep disorders (Stetter and Kupper, 2002). Even though breathing at RF does matter when considering the optimal means of endogenously achieving hfHRV while increasing BRS (Vaschillo et al., 2002), it is still not clear whether breathing at RF would help treat CWP (Downey and Zun, 2009).

Efferent parasympathetic activity to the heart (i.e., cardiac vagal tone) elevates during expiration relative to inspiration due to the central respiratory gating of vagal outflow (Eckberg, 2003) and stimulation of the baroreceptors (Stancák et al., 1991b; Jafari et al., 2017). Prolonged duration of the exhalation phase during DB causes cardiac vagal tone to increase along with hfHRV across the entire respiratory cycle (Strauss-Blasche et al., 2000). FMS patients who breathe at half their normal rate are able to decrease pain and depressive symptoms more than when they are breathing normally (Zautra et al., 2010). This strongly suggests that the vagus nerve may be a prime target when considering a central mechanism responsible for producing respiratory hypoalgesia in CWP.

## Central Mechanism: Vagal Nerve

The vagal nerve is the tenth cranial nerve composed of approximately 80% afferent fibers (which carry essential information from the body to the brain) and 20% efferent fibers (which send signals from the brain to the body) (Howland, 2014). Vagus nerve stimulation (VNS), which typically involves electrical stimulation of the vagal nerve, is an approved therapy for both refractory epilepsy and treatment-resistant depression (Howland, 2014; Vonck et al., 2014). Due to its central role in the bidirectional transmission and mediation of sensory information between the brain and the body (Howland, 2014), the vagus nerve may also be a promising mechanism for potentially treating the pathophysiology of CWP.

Experimental studies on animals (Ren et al., 1993, 1988) and preliminary intervention trials on humans (Lange et al., 2011; Busch et al., 2013) have shown that VNS can modulate multiple pathophysiological mechanisms inherent in CWP: VNS has shown to strongly reduce peripheral inflammatory cytokines in animals and in humans (Tateishi et al., 2007; Meregnani et al., 2011), decrease sympathetic tone by modulating descending serotonergic and noradrenergic neurons (Randich and Gebhart, 1992), decrease malondialdehyde (a biological marker of oxidative stress) (Pavithran et al., 2008), reverse pain-related brain activity patterns by reducing hippocampal and amygdala activity and increasing insular cortical and left prefrontal cortex activity (Kraus et al., 2007), and drive the antinociceptive effects of opioids and their derivatives (Tarapacki et al., 1992; De Couck et al., 2014).

VNS as a means of pain treatment has been traditionally administered through invasive procedures, known as invasive VNS (iVNS), which typically involve the surgical implantation of electrodes around the cervical vagus nerve (Chakravarthy et al., 2015). iVNS has a high risk for adverse events that include voice alteration, paresthesia, cough, headache, dyspnea, pharyngitis, and pain at the site of stimulation (Ben-Menachem et al., 2015). These adverse events often require a decrease in stimulation strength or even permanent deactivation and/or removal of the iVNS device. An effective non-invasive alternative to iVNS is transcutaneous VNS (tVNS). The tVNS system sends electrical impulses through the skin (transcutaneous) of the outer ear straight into the auricular branch of the vagus nerve (Peuker and Filler, 2002). Intensity, pulse duration, and frequency of the tVNS can be adjusted accordingly (Frangos et al., 2015).

Even though a number of studies using high intensity tVNS have not found any major side-effects, tVNS can still be accompanied by slight pain, burning, tingling, or itching sensations near the sight of the electrodes (Kraus et al., 2007; Dietrich et al., 2008). tVNS devices, like implantable VNS systems, are expensive to obtain, maintain, and have a narrow patient distribution (Howland, 2014). There is also no scientific consensus regarding the frequency and strength of tVNS stimulation for pain treatment (Chakravarthy et al., 2015) nor is there a clear understanding of how a constant pulse frequency mirrors endogenous vagal nerve activity (it likely does not communicate in consecutive 30 s intervals as most of the tVNS devices do) (Chapleau and Sabharwal, 2011).

Another important factor to consider is that the vagus nerve is not one uniform structure. It is instead composed of a diversity of molecularly distinct neuron types with different anatomical projections and functions (Chang et al., 2008). Artificial means to either transcutaneously or surgically stimulate the entire vagus nerve without cell specificity may be a main cause of unwanted side effects and lack of effect in CWP patients. Habituation (the loss of efficacy over time) or the appearance of new adverse events during chronic therapy limits VNS usefulness and should be assessed (Morris and Mueller, 1999). In addition, the risk, cost, and benefits of each type of vagal nerve–enhancing intervention, in relation to pain reduction and side effects, must be considered (De Couck et al., 2014). Therefore, DB as a means of endogenously stimulating the vagal nerve may be a better option for CWP patients.

Various forms of paced slow breathing have shown to influence brain electrical activity which may be mediated by VNS arising from the diaphragm (Stancák et al., 1991a, 1993). This cardio-respiratory stimulation of the vagus nerve may explain some of the overall positive emotional and cognitive benefits of DB (Howland, 2014). DB as a means of VNS may potentially decrease the pathophysiological processes involved in central sensitization as seen in CWP. This action may be the mechanism by which VNS reduces widespread musculoskeletal pain in FMS and other comparable pathologies (Lange et al., 2011; Chakravarthy et al., 2015). Among the many distinct neuron types within the vagus nerve are nerve fibers that specifically innervate the lungs and airways and which have been found to be vital for DB. These sensory neurons provide critical information needed to control respiration rate and regulate airway tone (Widdicombe, 2001; Canning et al., 2006). Within the airways, these vagal sensory neurons detect markers of inflammation, illness, and the mechanical stretch of the lungs during cycles of inhalation and exhalation while performing DB (Widdicombe, 2001; Carr and Undem, 2003).

Afferent vagal axons enter the brain bilaterally and primarily target the nucleus of the solitary tract (NTS), the first synapse in the baroreflex. This brainstem nucleus transmits sensory information to the limbic system and other deeper brain structures (Berthoud and Neuhuber, 2000; Kubin et al., 2006; Howland, 2014) and acts as an important interface between autonomic and regulatory centers within the brainstem and the central nervous nociceptive system (Bruehl and Chung, 2004; Duschek et al., 2013). This permits a central modulation of both cardiorespiratory and nociceptive activity (Chalaye et al., 2009; Jafari et al., 2017). The baroreceptor system connects the NTS with higher cerebral regions related to pain emotion, cognition, and autonomic control such as the periaqueductal gray, nucleus raphe magnus, locus coeruleus, anterior cingulate cortex, hypothalamus, and thalamus (Duschek et al., 2013). The periaqueductal gray’s involvement in central pain processing implies that local alterations within this region during DB may underlie a main component of the antinociceptive effects of VNS in humans (Kirchner et al., 2000; Henry, 2002; Subramanian and Holstege, 2010). The ability of VNS to reverse pain-related brain activity patterns during DB and target both affective and cognitive networks associated with pain raises the question as to whether a psychological mechanism could potentially amplify these reversal effects (Jafari et al., 2017).

Psychologically, pain can be perceived cognitively (as measured by the intensity of aching, burning, or stinging) (Turk and Rudy, 1992) and affectively (as measured by the unpleasantness of those sensations) (Frangos et al., 2017). Attentional modulation of pain preferentially affects perceived pain intensity, whereas the affective modulation of pain (dependent on one’s mood) preferentially modulates the unpleasantness of pain (Villemure et al., 2003; Loggia et al., 2008). This is highlighted by the fact that dissociable neural networks of attention and mood exist in regard to the modulation of pain intensity and unpleasantness (Legrain et al., 2009; Villemure and Bushnell, 2009). Even though pain intensity is frequently recommended as the primary indicator for determining intervention efficacy (Younger et al., 2009), it has been argued that pain intensity is not the best measure of the success of CP treatment (Ballantyne and Sullivan, 2015). Pain which is initially associated with the classic sensory “pain connectome” is later associated with brain regions involved in emotion and reward — over time, pain intensity becomes linked less with nociception and more with emotional and psychosocial factors (Hashmi et al., 2013; Ballantyne and Sullivan, 2015). The trending positive effects of VNS on various cognitive and affective processes are a further indication that psychological factors should be considered in studies and treatments investigating vagal pain modulation (Frangos et al., 2017).

## Cognitive Psychological Mechanism: Non-Directive Meditation

Meditation encompasses a broad family of complex emotional and attentional regulatory training regimes that can be roughly categorized into three separate groups dependent upon the type of attention being practiced. Focused Attention (FA) meditation entails the voluntary focusing of attention on a chosen object or stimulus (usually the breath). Whenever attention wanders away from the breath, the meditator tries to quickly detect mind wandering and gently, but firmly, brings their attention back to the physical sensation of the breath (Brewer et al., 2011). Open Monitoring (OM) meditation involves a non-reactive monitoring of the content of inner and outer experiences from moment to moment (Lutz et al., 2008b). During OM meditation, the practitioner pays attention to whatever comes into and out of awareness — whether it may be a thought, emotion, or body sensation — without holding onto it or changing it in any way (Brewer et al., 2011). Non-directive meditation is somewhat of a combination of the two: where the presence of spontaneously occurring thoughts, images, sensations, memories, and emotions is accepted without actively directing attention toward them (FA) or away from them (OM) (Ellingsen and Holen, 2008; Nesvold et al., 2012). A practitioner of ND effortlessly places a relaxed focus of attention on a mental or audible sound (such as the non-semantic sound of an inhalation and exhalation) while non-judgmentally allowing the focus of their attention to shift toward spontaneously occurring thoughts, images, sensations, memories, or emotions (Davanger et al., 2010).

Chronic widespread pain patients who report high pain intensity display cognitive deficits and show significantly impaired performance on cognitively demanding tasks when compared to CWP patients with low pain intensity and healthy controls (Eccleston, 1995; Hart et al., 2000). The difficulty for CWP patients to sustain task-relevant attention causes pain-related anxiety, pain hypervigilance, pain catastrophizing, and long-term cognitive distress (Sullivan et al., 1995; Crombez et al., 2005). Attention diversion and attention allocation (James, 2013) (two skills trained during FA meditation) have been shown to reduce pain-related anxiety, pain hypervigilance, and pain interference and increase executive functioning for patients diagnosed with several CP conditions (Elomaa et al., 2009). Experimental studies comparing the efficacy of OM meditation practiced with DB (OM-DB) and FA meditation practiced with DB (FA-DB) at the same respiration rates (7 cycles per minute) and depths (2 cm amplitude/cycle) show that OM-DB significantly increases cold and hot pain threshold and attenuates pain perception significantly more than FA-DB in healthy adults (Busch et al., 2012) and is accompanied by concomitant changes in cardiac activity similar to what is observed during DB (Chalaye et al., 2009). However, as pain transitions from acute to chronic, there is an accompanying neurobiological shift toward emotionally related circuitry within the brain (Hashmi et al., 2013). The transition of pain from a sensory, cognitive, and nociceptive state to becoming more of an emotional burden is reflected neurologically within the default mode network (DMN) of those suffering from CWP. This is imperative to consider when choosing a suitable meditation that can not only treat cognitive functioning and pain, like FA and OM, but also modulate affective functioning in CWP.

fMRI analyses show that among the five major resting-state networks, only the DMN consistently exhibits altered spatial extent and functional connectivity properties in those suffering from CP when compared to healthy controls (Baliki et al., 2014). The DMN participates in episodic memory (Zysset et al., 2002), the monitoring and detection of internal salient events (Raichle et al., 2001), and affective processing (Xu et al., 2014). DMN functional connectivity in patients with several CP conditions shows that as pain becomes chronic, the DMN increases coupling between pain-related regions and affective regions such as the insular cortex — a brain region that signals both the sensory and affective properties of CP (Apkarian et al., 2011; Baliki et al., 2014). Conversely, a reduction of the intrinsic DMN connectivity to the insula in FMS patients following 4 weeks of acupuncture was shown to be strongly correlated to reductions in pain (Napadow et al., 2012).

It has been suggested that abnormal DMN coupling and communication with other affective brain systems in CWP may be driven by attention to pain in daily life (Letzen and Robinson, 2017). Those who suffer from CP report that their attention to ongoing pain often varies (Viane et al., 2004) and that the intensity of their pain can fluctuate on short time scales (seconds/minutes) (Foss et al., 2006). These daily fluctuations of attention and pain intensity involve constant interactions between the DMN and the antinociceptive system — an interaction which may determine the course of pain-related structural brain reorganization and CP prognosis (Kucyi et al., 2013). A meditation that has shown to modulate attention to pain in respect to emotion is ND meditation.

ND meditation, which permits mind wandering, involves a more extensive activation of brain areas associated with episodic memories and emotional processing, than during FA meditation, OM meditation, or regular rest (Xu et al., 2014). Most mindfulness practices view mind wandering as a distraction and a gateway to rumination, anxiety and depression (Sood and Jones, 2013; Xu et al., 2014). These practices make it their goal to reduce mind wandering and its potentially negative consequences (Brewer et al., 2011; Sood and Jones, 2013). However, mind wandering and activation of the DMN during ND meditation may serve introspective and adaptive functions beyond rumination and daydreaming (Ottaviani et al., 2013) — especially for those with CWP.

Stronger structural connectivity between the periaqueductal gray and the DMN is associated with the ability to mind wander away from pain and thus treat it as a non-distractor (Kucyi et al., 2013). By engaging in ND meditation, patients with CWP could possibly stimulate and rewire the DMN by allowing thoughts, images, sensations, memories, and emotions related to their pain to emerge and pass freely without actively controlling, escaping, or pursuing them (Xu et al., 2014) — over time this may reduce stress by increasing awareness and acceptance of pain as an emotionally charged experience (Ellingsen and Holen, 2008; Lutz et al., 2008a; Sood and Jones, 2013). ND meditation may teach a CWP patient to increase their ability to accept and tolerate the stressful and emotional burden of ongoing pain during the meditation and also outside of it (Davanger et al., 2010).

Functional connectivity between the DMN and brain regions associated with emotion regulation (i.e., the insula and parahippocampus) in patients diagnosed with major depressive disorder (commonly co-morbid in those with CWP) has also been shown to decrease after 1 month of tVNS compared to sham stimulation. The change in depression severity significantly correlated with functional connectivity changes between the DMN and regions that are implicated in both pain modulation and emotion, such as the anterior insula and anterior cingulate cortex (Fang et al., 2016; Su et al., 2016). However, investigations of vagal pain modulation do not reliably report the preferential modulation of affect on pain unpleasantness even though behavioral and brain imaging studies show that tVNS improves affect and produces functional changes in brain regions where pain modulation and affect converge (Frangos et al., 2017). Investigations which combine DB (as a means of vagal innervation) and ND meditation may be able to display some of the psychobehavioral changes that can occur in patients with CWP.

A study (Mehling et al., 2005) compared the effects of a 6–8 week breath therapy intervention (a type of ND meditation combined with DB) and high-quality, extended physical therapy on pain, disability, and emotional wellbeing as expressed in diary entries for patients diagnosed with chronic low back pain (cLBP). Researchers found that the pre to post-intervention changes in standard low back pain measures of pain and disability were comparable in both groups. However, major differences between groups appeared for emotional effects as displayed in patient diaries. cLBP patients randomized to the ND breath therapy intervention had diary entries with emotionally richer insights about their pain and coping with stress with few or no entries in the physical therapy group’s diaries. Interestingly, the more gentle and breath-focused the physical therapy was, the more similar the emotional diary statements were to breath therapy such as: “calmness,” “less anxiety,” “sense of emotional strength,” “encouraged,” “uplifting,” and “more emotional awareness” (Mehling et al., 2005).

The affective interoception seen in these diary statements as a result of an ND and DB- based therapy intervention reflect the neurobiological activity seen in brain regions, such as the insula, which result from VNS (Critchley et al., 2004; Frangos et al., 2015). The anatomy of interoceptive processing indicates a convergence of signals derived from the spine and the vagus nerve which travel toward cortical representations within the insular cortex (Craig and Craig, 2009). Interoception seems to be dependent upon a combination of both anatomy and motivational content (Craig and Craig, 2009); physiological sensations, such as pain, and organ signals are carried centrally by afferents that mostly ascend the spinal laminar 1 spinothalamic tract. This suggests a dedicated interoceptive-motivational pathway (Critchley and Garfinkel, 2017).

## Affective Psychological Mechanism: Motivation

Preclinical and clinical evidence shows that tVNS simultaneously modulates both pain and mood, yet little is still known about possible indirect descending effects of altered mood states on pain perception for those suffering from CWP. Previous studies have shown that both positive and negative mood states can modulate the affective dimension of pain unpleasantness (Frangos et al., 2017). CWP patients are detrimentally affected by negative emotional states and attitudes that fluctuate on a daily basis which can exacerbate their pain symptoms (Haythornthwaite and Benrud-Larson, 2000; Schanberg et al., 2000; Frangos et al., 2017). Yet positive emotions, such as resiliency and optimism, can sustain CWP wellbeing and recovery (Ong et al., 2010; Sturgeon and Zautra, 2010) and may also be a promising means of treating the inflammatory etiology of CWP (Kox et al., 2014; van Middendorp et al., 2016). Due to the fact that systemic low-grade inflammation is associated with CWP (Gerdle et al., 2017), utilizing motivation and positive treatment expectancy may by an important treatment factor to consider. Therefore, identifying the interoceptive factors that influence pain coping and positive treatment expectancies could potentially help clinicians facilitate the use of adaptive coping strategies for treating CWP patients (Jensen et al., 1991).

Patients with CWP show significant anatomical and functional changes within reward/motivational circuitry within the brain that strengthen emotional and affective pain mechanisms (Apkarian et al., 2009; Apkarian et al., 2013). These maladaptive changes in aversive/motivational circuits are a challenge for CWP treatment (Navratilova and Porreca, 2014). However, targeting reward/motivation circuits can be a source for treatment that may provide a path for normalizing the neurobehavioral consequences of CWP and help surpass symptomatic management (Navratilova and Porreca, 2014).

Pain can be considered a homeostatic emotion (Craig and Craig, 2009) (such as hunger, thirst, or the desire to sleep) — a mechanism which involves receptors that detect internal imbalances (i.e., sensations) and aversive emotions that demand a behavioral response (i.e., motivation) to ensure the organism takes proper action to restore homeostasis (Denton et al., 2009). Therefore, pain can produce a strong motivational drive which promotes escape or, in the case of CWP, seek relief (Craig and Craig, 2009). Due to their fundamental role in survival, the basic neurological networks of reward, expectation, and motivation have evolved early and are conserved across species (Andreatta et al., 2012). The evolutionary role of negative (pain) and positive (relief) affective states is to elicit motivations that typically result in escape/avoidance and approach behaviors (Craig and Craig, 2009) — this allows an individual in pain to learn how to predict painful or relieving situations and/or triggers in the future (Wiech and Tracey, 2013). Even though ‘pain relief as reward’ has been a driver for human survival and wellbeing (Leknes et al., 2011), the constant daily demand for pain relief in those suffering from CWP can actually lead to the suppression of other emotions (i.e., natural rewards). This in turn can potentially lead to anhedonia (reward deficit state) and diminished quality of life (Simons et al., 2014).

Multilevel modeling analyses (Zautra et al., 2005) indicate that weekly elevations of pain and stress predict increases in negative affect in patients with CWP/FMS and that greater positive affect predicts lower levels of pain. Research investigating the relationships between CP patients’ dispositional optimism and pessimism and the coping strategies they use has found that there is a positive relationship between optimism and the use of active coping strategies (i.e., handling the pain or carry on functioning despite the pain) and pessimism and the use of passive coping strategies (i.e., giving control over pain to another person or allowing pain to adversely affect other areas of the patient’s life) (Ramírez-Maestre et al., 2012). Active coping is associated with low levels of pain, anxiety, depression and impairment and high levels of functioning whereas passive coping is related to high levels of pain, anxiety, depression and impairment and low levels of functioning for those suffering from a variety of CP conditions including CWP (Lin and Ward, 1996; Ramírez-Maestre et al., 2012). If left untreated, the neurologically affective quality of CWP has the potential to increase emotionally related pain catastrophizing which can predict the future onset of CP severity (Picavet et al., 2002; Sullivan et al., 2001a,b) and increase pro-inflammatory cytokines (Koch et al., 2007).

Emerging evidence suggests that systemic low-grade inflammation is associated with CWP and that the presence of inflammation could promote the spreading of pain, a hallmark sign of CWP (Gerdle et al., 2017). A multivariate, explorative, cross-sectional study (Gerdle et al., 2017) found that eleven pro-inflammatory proteins are significantly differentiated between healthy controls and CWP patients. Positive significant correlations exist between several proteins and pain intensity in CWP patients (Gerdle et al., 2017). However, positive optimism-inducing expectancies about health can induce immune responses that may directly and positively influence health and treatment outcomes for CWP. Motivation and expectation play major roles in the treatment outcomes for a wide variety of immune-mediated conditions and are shown to strengthen or mimic the effects of regular long-term therapies (van Middendorp et al., 2016).

The psychological and emotional state of an individual can have a significant impact on pain perception (Craig and Craig, 2009; Wiech and Tracey, 2009; Bushnell et al., 2013). Predictions about future (expected) pain or relief have shown to significantly influence the actual pain or analgesia that is experienced. Positive treatment expectancy produces heightened analgesia (placebo analgesia) (Atlas and Wager, 2012) while negative expectancy may exaggerate pain (nocebo) (Tracey, 2010; Doering and Rief, 2012). BOLD-fMRI measurements taken of the human spinal cord demonstrate that expectation of pain relief (placebo analgesia) directly reduces nociceptive processing in the dorsal horn of the spinal cord, presumably via intrinsic descending inhibitory mechanisms (Craig and Craig, 2009; Eippert et al., 2009). A proof-of-principal study (van Middendorp et al., 2016) also found that generalized outcome expectancy optimism is a potential determinant of the autonomic and immune response to an intravenously administered bacterial endotoxin (2 ng/kg Escherichia coli endotoxin) in healthy subjects after a short-term training program consisting of meditation, breathing exercises, and cold exposure.

A higher degree of optimism in subjects randomized to a training program consisting of meditation, breathing exercises, and cold exposure was associated with profoundly higher plasma epinephrine levels during the intravenous administration of the bacterial endotoxin followed by a more rapid and profound increase in anti-inflammatory IL-10 levels when compared to those in the non- trained group. A more positive expectation of the effects of the meditation, breathing, and cold exposure training on the psychophysiological reaction to the bacterial endotoxin was associated with lower flu-like clinical symptoms and lower subsequent pro- inflammatory TNF-α, IL-6, and IL-8 levels than the non- trained group (van Middendorp et al., 2016). The researchers noted that the DB breathing techniques practiced by the trained individuals mainly accounted for the increase in epinephrine and subsequent attenuation of the inflammatory response (Kox et al., 2014). Inflammatory conditions that produce or are induced by pain may also improve with DB vagal nerve innervation as descending vagal signals activate anti-inflammatory pathways that suppress secretion of pro-inflammatory cytokines during tVNS (e.g., TNFα and IL-1 IL β) (Borovikova et al., 2000), which could subsequently ameliorate associated pain (Yuan and Silberstein, 2016; Frangos et al., 2017).

The signaling of systemic inflammation is communicated to the brain via neural (predominantly vagus nerve) pathways, humorally via circulating cytokines, and directly via immune cells (Zaki et al., 2012). Chronic states of inflammation, as seen in CWP (Gerdle et al., 2017), influence emotion through a coordinated set of motivational changes conceptualized as ‘sickness behaviors’. These behaviors include fatigue, anhedonia, social withdrawal and irritability— all symptoms which are commonly shared with depression (de Heer et al., 2014; Harrison, 2016)and seen in CP patients (Harris, 2014). Subjective interoceptive experience which is generated by ND meditation and DB may help CWP patients generate new predictions and expectations about information (i.e., pain) coming from inside the body (Pacheco-López et al., 2006; Critchley and Garfinkel, 2017). Efferent (i.e., top-down) predictions concerning the state and outcome of the body (i.e., “I will feel calmer and less pain from this treatment”) is expressed in the autonomic nervous system, in endocrine, and in immune responses (Critchley and Garfinkel, 2017) which can beneficially effect a patient’s peripheral physiology (Seth, 2013; Seth and Friston, 2016). In turn, emotions and feelings arise through the interaction of descending bodily predictions (i.e., “I will feel calmer and less pain from this treatment”) through autonomic drive and ascending prediction errors (i.e., chronic stress and pain).

Evaluating pain-motivated behaviors can provide a path for the assessment of new treatment efficacy for CWP with a high likelihood of translational relevance. Due to the current notion that CWP may partly be an immune-mediated condition, this evidence raises the question as to whether motivation and expectation should be targeted and implemented within CWP treatment, especially in regards to a ND meditation and DB intervention.

## Motivational Non-Directive Resonance Breathing: From Theory to Practice

In order to test whether MNRB is an effective psychophysiological intervention for endogenously treating pain intensity and disability in those who suffer from CWP, a randomized controlled clinical trial (Clinical Trials Identifier: NCT03180554) lead by the lead author (C.E.P.) and co-author (H.B.J.) in the Spring of 2019 will compare and investigate the treatment efficacy of MNRB and tVNS on patients diagnosed with CWP.

Consenting CWP patients (*N* = 112) who are referred to the Department of Pain Management and Research at Oslo University Hospital, Ullevål, in Oslo, Norway, will be randomized into one of four independent groups. Half of these participants (*N* = 56) will be randomized to either an experimental tVNS group or a sham tVNS group. The other half (*N* = 56) will be randomized to either an experimental MNRB group or a sham MNRB group. Both experimental and sham treatment interventions will be delivered twice per day at home, 15 min/morning and 15 min/evening, for a total duration of 2 weeks. Participants are invited to the clinic twice for pre- and post-intervention data collection. The primary outcomes are changes in photoplethysmography measured HRV and self-reported average pain intensity measured by the numeric rating scale. Secondary outcomes include changes in pain detection threshold, pain tolerance threshold, and pain pressure limit determined by computerized pressure cuff algometry as well as blood pressure and health related quality of life.

Participants randomized to the experimental or sham MNRB treatment will utilize an innovative smartphone-based program called *MNRB* and a CE-approved respiratory gating device called BarTek^TM^ designed by the lead author (C.E.P.) and engineered by Dr. Marcin Czub at the University of Wrocław in Wrocław, Poland. The *MNRB* program and BarTek^TM^ device work in-sync in order to deliver and guide CWP patients in both the sham and experimental versions of MNRB. The BarTek^TM^ respiratory gating device is attached to an elastic strap which is placed around the patient’s abdomen, below the rib cage and an inch above the navel (behind which the thoracic diaphragm is located) (Bains and Lappin, 2018). Throughout the MNRB session, a strain gauge circuit accurately measures the tension produced during RF breathing. Respiration frequency and depth are calculated with a high resolution analog digital converter within the BarTek^TM^ device and transmitted to the *MNRB* smartphone program via Bluetooth.

MNRB is to be practiced at home, twice a day for 2 weeks, in a relaxed semi-Fowler position (30 degree tilt from the horizontal) with feet flat on the floor, hands on thighs, and palms facing upward. Ideally, MNRB should be practiced with a head tilt no more than 30 degrees from the horizontal (Mukai and Hayano, 1995) due to the fact during high-level tilt (30–90 degrees), the R-R interval and hfHRV progressively decrease with tilt angle (*P* < 0.001 for both) (Berna et al., 2014; Quintana et al., 2016). This relaxed sitting position is also conducive for patients who will be taking a 1-min HRV recording (Laborde et al., 2017) immediately before and after each MNRB session.

When in position, CWP patients strap the BarTek^TM^ respiratory gating device around their abdomen, open the MNRB program on the smartphone, and are guided (Kniffin et al., 2014) from an average respiration rate of 12 breadths/min to a RF of 6 breadths/min. The average respiratory rate for a healthy adult at rest is typically defined as 12–20 breadths/min (Cleveland Clinic, 2018b) whereas patients diagnosed with FMS have shown to have a respiration rate of around 13.68 breadths/min (Zautra et al., 2010). Participants are instructed to use the diaphragm to breathe in slowly through the nose to full inspiratory capacity and exhale to full expiratory capacity through pursed lips by tightening and pulling the stomach back toward the spine. Participants are instructed to retain a 1:2 inhale: exhale ratio in order to efficiently increase hfHRV across the entire respiratory cycle (Strauss-Blasche et al., 2000) and retain their breadth after full inhalation and full exhalation. Inclusion of a post-exhalation rest period significantly decreases HR (*p* < 0.001) and increases hfHRV (*p* < 0.05) (Russell et al., 2017). Participants are further instructed not to move or to speak and to allow the chest to remain immobile throughout the entirety of the session (Mosby, 2009; Cleveland Clinic, 2018a).

The MNRB session begins with a transition period during which patients are taken from an average respiration frequency of 12 breadths/min to a RF of 6 breadths/min. During this transition period, patients are to attend to a respiration guide while listening to a 110 Hz frequency which has been shown to increase hfHRV (Hori et al., 2005). While breathing with the respiration guide, participants are also provided with a series of written sentences that appear and disappear on the smartphone screen. These sentences are of an affective and motivational tone which encourage patients to reason about emotional issues that may surround and define their pain.

Emotional information derived from their MNRB practice can help CWP patients to become motivated to solve problems and achieve goals on a daily basis. In turn, this may be helpful for increasing emotional intelligence (Mayer J.D. et al., 2008). Emotional intelligence is highly predictive of important aspects of social/interpersonal functioning and professional success (Brackett et al., 2006; Moslehi et al., 2015). This can potentially benefit CWP patients in particular who suffer from high rates of long-term sick leave (Mose et al., 2016) and lack interpersonal skills (Hayaki et al., 2016). The adaptive use of emotional intelligence to become motivated and achieve goals in regards to increasing ones sense of emotional resilience and interoception requires the integration of many capacities that include: self-awareness, subjective perceptions, reasoning, and skilled behavioral responses (Killgore et al., 2017).

When CWP patients arrive at their target RF of 6 breadths/min following the transition period, the *MNRB* screen begins to darken and the respiratory guide disappears while the inhale/exhale sound guide along with the 110 Hz background frequency remains. At this moment, patients are invited to close their eyes and engage in a ND state of mind. Cardiac parasympathetic nervous activity indicated by hfHRV has been found to increase more while listening to a 110 Hz sine wave when the eyes are closed as compared to when they are open (Hori et al., 2005). As described previously, patients are to engage in a flexible and nonjudgmental cognitive state between the sensation of breathing and any spontaneous stimuli which may arise moment by moment; attention is permitted to shift toward and away from spontaneously occurring thoughts, feelings, and sensations related or unrelated to their pain, and back to the repetition of the inspiration/expiration sound. This is unlike standardized mindfulness practices where sustained attention is required to maintain focus on the breath while cognitive control is required to detect mind wandering (Moore et al., 2012).

If the patient continues to correctly breathe at the RF of 6 breadths/min the respiration inhale/exhale sound guides silence and the patient is only left with listening to the 110 Hz auditory background tone. Continuing to breathe at RF will also cause this background tone to dissipate after a few breaths leaving the patient in complete silence. This teaches and entrusts the patient to embody the treatment on their own terms which in turn may lead to a sense of mastery over a 2 week period. Considering the high costs for running contemplative research trials, the type of meditation practice under investigation, and the multiple outcomes being explored, it is important to reevaluate the typical 4–8 week intervention duration of typical mindfulness-based meditation practices for treating specific psychophysiological parameters in CWP patients.

Most researchers hold the assumption that meditation practice has its effects in a cumulative way through long-term practice. However, current research (Zeng et al., 2017) shows that short-term influences of meditation practice have a more promising effect upon clinical outcomes and that continual meditation practice may not be necessary for maintaining effects (Cohn and Fredrickson, 2010). Therefore, this study will employ a 2-week meditation intervention where both short-term (i.e., daily) and long-term changes (i.e., changes in pre- to post-intervention measures) of self-reported pain and HRV effect patterns will be analyzed in accordance with the three R procedure: resting (i.e., pre-intervention), reactivity (i.e., tVNS/MNRB treatment), and recovery (i.e., post-intervention) (Stein and Pu, 2012). Following this procedure will aide in determining the differential treatment effects of experimental versus sham MNRB.

Trials have attempted but failed to design sham breathing procedures for control groups simply due to the fact that the influence of the diaphragm muscle cannot be ruled out (Kapitza et al., 2010; Eherer et al., 2012). However, the BarTek^TM^ device will allow us to see whether or not a participant randomized to the sham MNRB group has been utilizing their diaphragm during each treatment session. Participants in the sham MNRB group will practice MNRB with the same posture and time protocol as the experimental group (Chan et al., 2007; Russell et al., 2014). However, participants are instead instructed to breathe at the normal respiration rate for an adult (12 breadths/min) (Barrett and Ganong, 2013) by attentively following the visual respiratory pacer (Elstad, 2012) on the *MNRB* program while counting their breadth (Juel et al., 2017). There is no background frequency of 110 Hz playing during the sham MNRB session and only one sentence that remains on the screen for the entirety of the session which instructs participant to relax while breathing with the visual respiratory pacer indicated by the moving orb. Sham MNRB does not promote endogenous mastery of the treatment and remains on the screen for each treatment session.

## Conclusion

Contemplative research is challenged to evaluate, measure, and explain the effects of meditation and other contemplative practices on health and well-being when compared to mainstream treatment regimens. Even though these practices are of great interest to the scientific and medical communities within the field of CWP research and management, objective measures to best assess their beneficial outcomes are lacking (Desbordes et al., 2014). Current findings on the effects of meditation practice are few and inconsistent for various chronic pain conditions— studies suffer from inconsistencies within intervention deliverability and type while other’s show relatively few associations between outcome variables and the amount of meditation practice (Zeng et al., 2017). Previous theories which include the neurovisceral integration model, the polyvagal theory, the biological behavioral model, the RF model, and the psychophysiological coherence model provide important insights in regards to how vagal tone is important to consider when optimizing psychophysiological health. However, these theories lack a formalized and integrated means of methodologically applying the theory to practice within the field of CWP treatment.

Clinical and self-report pain intensity is often used as the sole primary outcome for determining intervention efficacy in clinical trials employing mainstream and alternative treatment interventions for those suffering from CWP (Williams et al., 2012). Yet it has been argued that pain intensity may not the best indicator of effective CWP treatment (Ballantyne and Sullivan, 2015). Based upon this conjecture and research showing a strong association between high pain intensity and low HRV readings in CWP patients (Barakat et al., 2012) we have chosen HRV as our primary outcome of interest along with self- report pain intensity. This will provide us with a more robust evaluation of our medical hypothesis and reveal the autonomic, respiratory, circulatory, endocrine and mechanical influences of MNRB on pain over both a short and long-term time frame.

The current lack of mainstream and alternative treatment efficacy, safety, and reliability calls for the development of new treatment modalities, such as MNRB, that meet the biopsychosocial needs of those suffering from CWP. Motivational ND resonance breathing (MNRB) could be an innovative, effective, and noninvasive means of CWP treatment. To our knowledge, we are the first to propose such an intervention that could potentially target CWP etiological factors we believe to be imperative for successful treatment.

## Author Contributions

CP is the lead and corresponding author for this work, responsible for the original conception and design of the chronic widespread pain treatment program presented and motivational ND diaphragmatic breathing. HJ interpreted and analyzed the biopsychosocial framework and psychological theory of the program in respect to chronic widespread pain treatment. CP and HJ revised the work critically for important intellectual content, approved the final version to be published, and are accountable for all aspects of the work in ensuring that questions related to the accuracy or integrity of any part of the work are appropriately investigated and resolved.

## Conflict of Interest Statement

The authors declare that the research was conducted in the absence of any commercial or financial relationships that could be construed as a potential conflict of interest.
